# Human Collective Intelligence under Dual Exploration-Exploitation Dilemmas

**DOI:** 10.1371/journal.pone.0095789

**Published:** 2014-04-22

**Authors:** Wataru Toyokawa, Hye-rin Kim, Tatsuya Kameda

**Affiliations:** 1 Department of Behavioral Science, Hokkaido University, Sapporo, Hokkaido, Japan; 2 Japan Society for the Promotion of Science, Tokyo, Japan; 3 Center for Experimental Research in Social Sciences, Hokkaido University, Sapporo, Hokkaido, Japan; Durham University, United Kingdom

## Abstract

The exploration-exploitation dilemma is a recurrent adaptive problem for humans as well as non-human animals. Given a fixed time/energy budget, every individual faces a fundamental trade-off between exploring for better resources and exploiting known resources to optimize overall performance under uncertainty. Colonies of eusocial insects are known to solve this dilemma successfully via evolved coordination mechanisms that function at the collective level. For humans and other non-eusocial species, however, this dilemma operates within individuals as well as between individuals, because group members may be motivated to take excessive advantage of others' exploratory findings through social learning. Thus, even though social learning can reduce collective exploration costs, the emergence of disproportionate “information scroungers” may severely undermine its potential benefits. We investigated experimentally whether social learning opportunities might improve the performance of human participants working on a “multi-armed bandit” problem in groups, where they could learn about each other's past choice behaviors. Results showed that, even though information scroungers emerged frequently in groups, social learning opportunities reduced total group exploration time while increasing harvesting from better options, and consequentially improved collective performance. Surprisingly, enriching social information by allowing participants to observe others' evaluations of chosen options (e.g., Amazon's 5-star rating system) in addition to choice-frequency information had a detrimental impact on performance compared to the simpler situation with only the choice-frequency information. These results indicate that humans groups can handle the fundamental “dual exploration-exploitation dilemmas” successfully, and that social learning about simple choice-frequencies can help produce collective intelligence.

## Introduction

Maximizing the sum of rewards from several choice options, whose distributions are initially unknown and whose profitability may differ, is a ubiquitous adaptive problem for organisms across many taxa. Although such problems characterize many foraging situations in natural environments for humans as well as non-human animals (e.g., optimal prey choice), they are perhaps best illustrated by the situation that a gambler faces at a row of slot machines in Las Vegas, giving them their name: “multi-armed bandit” (MAB) problems [1]. When played, each machine yields a random reward from a distribution unique to the machine. The gambler has only partial, if any, knowledge about the properties of the machines at the outset, yet she/he may become better informed as time passes. Given a fixed budget and time, the gambler's task is to decide which machines to play and how many times to play each machine so that she/he can maximize the total reward earned over a sequence of plays [2]. As illustrated above, the core of the MAB problem lies in the trade-off between exploiting the option that has yielded the largest cumulative payoff so far and exploring the other options to acquire more information about their expected payoffs. The decision maker must strike an optimal balance between the two opposing actions to maximize the overall profit.

Given the ubiquity of the exploration-exploitation dilemma, the MAB problem has attracted attention across many disciplines, including operations research [3], information science [4], statistics [5], [6], economics [7], biology [8], and psychology [9]. Most previous research has focused on identifying optimal or approximate strategies for individual learners to solve this dilemma [2], [10]–[12] and comparing actual human performance with such normative strategies in the context of individual reinforcement learning [13], [14].

Interestingly, however, natural MAB problems are often solved collectively by a group of agents. For example, eusocial insects such as ants and honeybees often show excellent performance in foraging – being able to locate and exploit the best of several resource patches with different qualities [15]–[17]. Even though these animals are limited in terms of learning ability and explore only one or two sites individually, they seem to solve the exploration-exploitation dilemma collectively by evolved coordination mechanisms that function at the colony level [18], [19]. Such collective intelligence might also be expected from humans (as in the “wisdom of crowds” [20]–[23], but see [24]), especially given the availability of modern information technologies. Yet, in contrast to eusocial insects whose reproductive success is dependent on the success of their colony, human collective performance may suffer from free-rider problems [25], [26]. In groups involving non-kin members, free-riders who let others explore for better alternatives while exploiting their findings through social learning (“information scroungers”) are expected to appear frequently [27], [28], and may consequently undermine the benefits of collective intelligence [29]–[34]. In other words, for humans and other non-eusocial species, the exploration-exploitation dilemma exists not only at the within-individual level, but also at the between-individual level as a public-goods game [25], [35]. So how do humans solve the MAB problem in face of such dual dilemmas?

We address this question using a laboratory experiment. In the modern human environment, it is often argued that the MAB problem may be solved collectively by mass information-sharing systems [36]. For instance, with the growth of online shopping sites like Amazon or review sites like Yelp, consumers can learn how other consumers have evaluated products (e.g., Amazon's 5-star rating system), as well as how many others have purchased those products. Do such information-sharing systems in fact help us solve the MAB problem? And, if so, how do the two types of social information (frequency information in conjunction with evaluation information) affect our decisions?

Although several previous studies have demonstrated that consumer choices are actually influenced by social information on the Internet [37], [38] and in the MAB task [39]–[41], they did not directly assess how social information could improve the objective qualities of people's choice behaviors above and beyond individual choices. We thus implemented a test-bed for the MAB problem in the laboratory.

**Figure 1:**
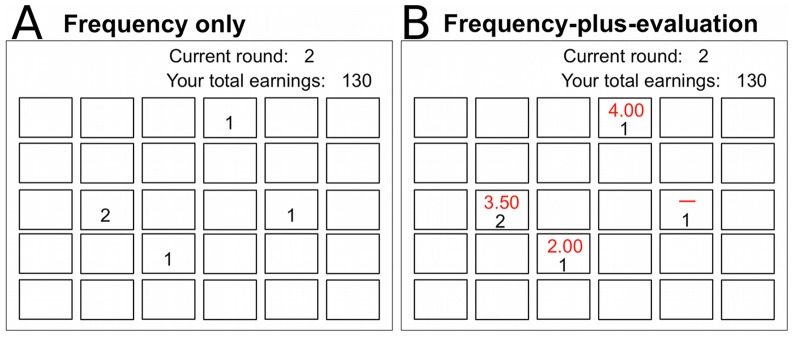
Example displays of choice stage. Except for round 1, social information about all 5 group members' choice behaviors in the preceding round was made available to each participant. A: An example display in the frequency-only sub-condition. The number displayed in each box (i.e., option) indicates how many members chose that option in the preceding round (round 1 in this example). B: An example display in the frequency-plus-evaluation sub-condition. In addition to the social-frequency information (lower black numbers), participants were informed of average evaluations (upper red numbers) that members had provided about their chosen options in the preceding round, on a 5-point scale that ranged from 1 (very bad) to 5 (very good). Evaluating options was not mandatory, and a horizontal red bar indicated that no evaluation was contributed about the option.

In the current experiment, a group of 5 participants worked on a 30-armed bandit problem (see Figure 1; also see Figure S1 and Figure S2 for details). Participants worked on the MAB task for a total of 100 rounds (though the number of rounds was not specified in advance). In each round, participants chose one of the 30 alternatives individually, receiving associated payoffs as personal rewards. For each chosen option, payoffs were randomly generated from a stationary uniform probability distribution. To simulate foraging environments in which patch quality is negatively correlated with frequency (i.e., high quality patches are rare), one of the 30 options was made to have the highest mean (the “category 6” option), followed by two “category 5”, three “category 4”, five “category 3”, eight “category 2”, and eleven “category 1” options (see Methods section for details about these categories). In addition to their own private payoff-feedback, participants could observe information about other members' choice behaviors from the preceding round. Within the group condition, we created two sub-conditions in which the richness of the social information was varied: (A) the “frequency only” sub-condition where individuals were informed of how many members had chosen each of the 30 alternatives in the preceding round; and (B) the “frequency plus evaluation” sub-condition where participants could rate their chosen option in each round on a 5-point scale (see Figure 1B) and could learn averages of those ratings for each option in addition to the social-frequency information. Rating the chosen option was entirely optional, and participants could skip the evaluation and proceed to the next round (see Methods section for details). As a baseline, we also ran an individual condition where participants worked on the MAB task alone without any social information. Our focus is thus on how the availability of social information may affect human collective performance on the MAB task in the face of within- and between-individual exploration-exploitation dilemmas.

## Results

In the following, we first examine whether participants benefited from social information to yield “collective intelligence” at the group level. We then examine how the richness of social information affected collective performance by comparing the frequency-only and the frequency-plus-evaluation sub-conditions.

### Group versus individual performance

**Figure 2:**
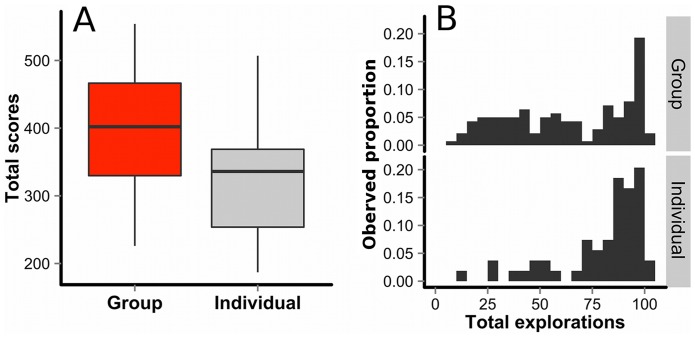
Comparison between individual and group conditions. A: Participants' scores in the individual and group conditions (N = 54 and N = 140, respectively). The y-axis refers to total scores of participants summed over 100 rounds, ranging from 100 (always choosing one of the 11 lowest-quality options) to 600 (always choosing the highest-quality option). The horizontal bars represent the median of each condition, while the boxes represent 50% ranges, and the upper and lower whiskers represent the highest and lowest values respectively that are within 1.5 * IQR (the inter-quartile range). B: Histograms of participants' exploratory choices among the 100 rounds in the individual and group conditions (N = 54 and N = 140, respectively). The x-axis refers to the total number of “exploratory” choices (when a participant selected options other than the one that had yielded the largest cumulative payoff in her/his own experience so far) out of 100 rounds. The y-axis refers to observed proportions of participants with a given exploratory-choice frequency in each condition.

Figure 2A shows box-plots of participants' cumulative scores over 100 rounds in the individual and group conditions. Participants' choices in each round were assigned scores ranging from 1 (choosing one of the 11 lowest-quality options) to 6 (choosing the single highest-quality option). Because participants in the group condition were nested in the same 5-person groups, we used a hierarchical linear model to analyze their individual performances (see Methods section for details). On average, participants achieved higher scores in the group condition (*M* = 397.3) than in the individual condition (*M* = 321.7), *ΔD* (difference between model deviances)  = 19.50, *p* = 1.0 * 10^−5^.

Figure 2B shows frequency distributions of how often participants engaged in exploratory choices out of the 100 rounds in the individual and group conditions. Here, exploration was defined as a choice in which a participant selected options other than the “greedy” option [10] that had yielded the largest cumulative payoff so far in her/his own experience (see Methods section for details). We estimated per-individual exploration probability by a hierarchical Bayesian logit model with the Markov Chain Monte Carlo (MCMC) method (see equation S1 in the Supporting Information). The 95% Bayesian credible interval of the fixed effect for the conditions (individual vs. group) was [−1.63, −0.27], which indicates that participants engaged in significantly fewer exploratory choices in the group condition than in the individual condition (the full results are shown in Table S1). Together with the results from Figure 2A, this implies that social information about other members' choices in the preceding round reduced each participant's total exploration time, helping her/him to exploit better options for a longer time in the group condition than in the individual condition. In other words, the within-individual exploration-exploitation dilemma was more efficiently resolved in the group condition than in the individual condition.

**Figure 3:**
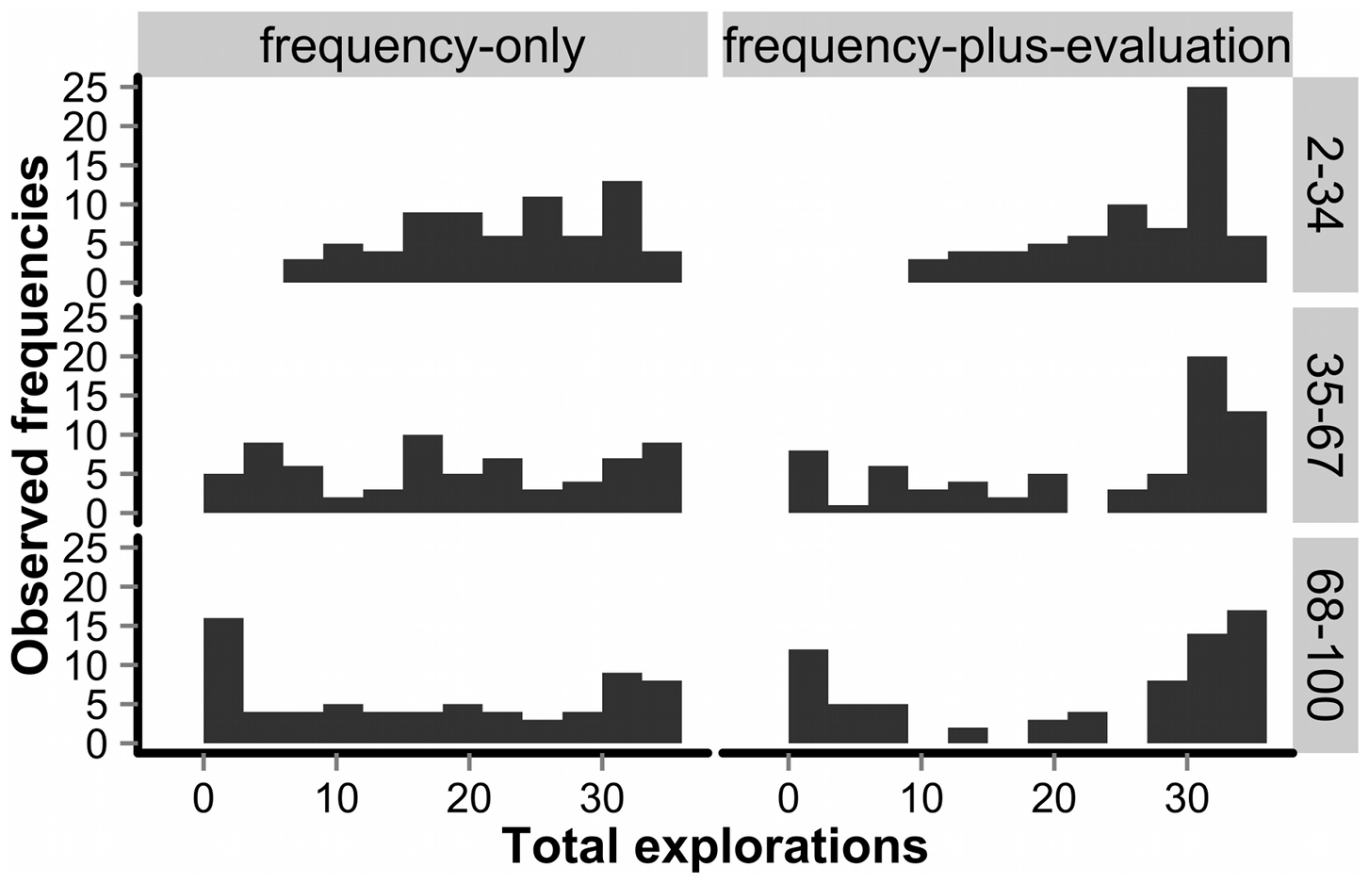
Temporal changes in frequency distributions of exploratory choices in the group condition. Shown from top to bottom, the first block (rounds 2–34), the second block (rounds 35–67) and the last block (rounds 68–100). The y-axis refers to observed frequencies of participants with a given exploratory-choice frequency in each sub-condition (both N = 70).

Interestingly, regarding the between-individual dilemma, the exploratory costs essential for emergence of collective intelligence (i.e., efficient collective exploitations of better options in the group condition) were not borne equally by all group members. Figure 3 displays participants' exploratory choices over the course of the experiment separately for each group sub-condition. We classified the experimental rounds (except for the 1st round where any choices were counted as “exploration”) into three blocks of 33 rounds each, and examined how often participants in the group condition engaged in exploratory choices in each block. As the figure shows, the frequency distributions changed over time, approaching a more U-shaped pattern in the later blocks. This U-shaped pattern, as well as the prevalence of explorations in groups, was more evident in the frequency-plus-evaluation sub-condition than in the frequency-only sub-condition (the 95% Bayesian credible interval of the fixed effect for the sub-condition in individual exploration probability was [0.07, 1.78]; see equation S2 and Table S2 for details). The observed U-shaped pattern implies that participants were divided largely between “information producers” who engaged in exploratory choices most of the time and information scroungers who free-rode on those efforts, exploiting others' findings through social learning [30]-[32]. Indeed, consistent with results from a recent social-learning-strategies tournament [42], participants who engaged in more exploratory individual learning attained smaller net profits within each 5-person group (*r*(140)  = −0.52, *p* = 0.45 * 10^−11^; see Figure S4). Yet, it is noteworthy that, despite the emergence of information scroungers, groups still benefited overall from exchanging social information about each other's behavioral choices in the MAB task [32], [42].

### Frequency-only versus frequency-plus-evaluation sub-conditions

Next we examine how enriched social information (i.e., evaluation information alongside frequency information) affected the collective performance of participants in the MAB task. A simple statistical intuition suggests that richer information (i.e., more predictors) should lead to better collective performance, as instantiated in many websites posting updated customer reviews of products in addition to sales volume or rank (e.g., Amazon or Yelp). However, this intuition may not necessarily hold for the group MAB situation. First, given the exploration-exploitation dilemma operating at the between-individual level (see Figure 3), some people may intentionally abuse the evaluation opportunity. For instance, they may rate options randomly, or even evaluate good options as “bad” in order to manipulate others into exploring different options while they take advantage of their own new findings. Second, if participants use the rating scale differently from each other, the evaluation information could be a statistically less reliable signal as compared to the frequency information, which is free from such interpersonal scaling differences. In short, the evaluation information may be susceptible to strategic manipulations as well as individual biases in scale-use.

**Figure 4:**
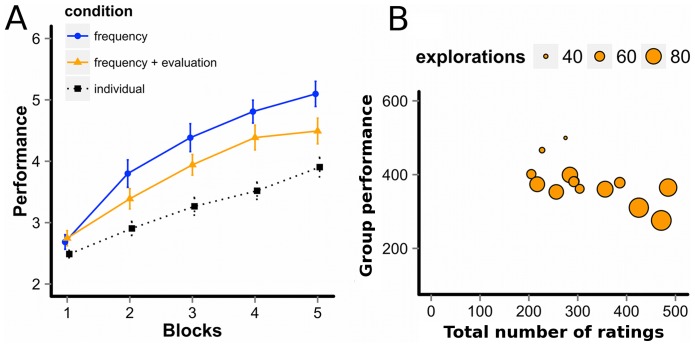
Effects of adding social evaluation information. A: Trajectories of participants' average scores. The scores of all 5 members of each group were averaged to yield aggregated group performance scores in each block that could range from 1 (choosing the lowest-quality options) to 6 (choosing the highest-quality option). Thus, the unit of analysis was the group, with N = 14 in each sub-condition. Means of these group scores were plotted for the 2 sub-conditions and 5 blocks in the figure. As a baseline, average scores of participants in the individual condition were also plotted (N = 54). The error bars indicate ±1 SEM. B: Scatter plots of collective scores against the total number of ratings contributed and the total number of explorations in groups of the frequency-plus-evaluation sub-condition (N = 14). The x-axis refers to the total number of times group members chose to rate their payoffs over the 100 rounds (minimum: 0, maximum: 500), and the y-axis refers to the group's overall score (minimum: 100, maximum: 600). The size of circle represents the total number of exploratory choices in the group (minimum: 0, maximum: 500– see Fig. 2 for the definition of exploration).

Figure 4A shows the trajectories of participants' average scores in the frequency-only and frequency-plus-evaluation sub-conditions (as a comparison, the trajectory in the individual condition was also displayed). For this analysis, we divided the 100 rounds into 5 equal blocks. A 2 (Sub-conditions) ×5 (Blocks) repeated-measures Analysis of Variance (MANOVA) yielded a main effect of blocks (*F*(4, 26)  = 114, *p* = 3.0 * 10^−6^) and an interaction effect (*F*(4, 26)  = 2.53, *p* = 0.0448), while a main effect of sub-conditions was not significant (*F*(1, 26)  = 2.67, *p* = 0.11). As can be seen in Figure 4A, performance generally improved over time, but the improvement was smaller in the frequency-plus-evaluation sub-condition than in the frequency-only sub-condition (see also Figure S3 for trajectories of all participants' performances). A post hoc Shaffer's multiple comparison test revealed that, in block 5, the average score was significantly lower in the frequency-plus-evaluation sub-condition (*M* = 4.49) than in the frequency-only sub-condition (*M* = 5.10; *F*(1, 26)  = 4.27, *p* = 0.0488), which suggests that, contrary to the aforementioned intuition, the additional evaluation information may have had a detrimental effect on participants' performance in the group MAB task.

To shed some light on the potentially detrimental effects of the evaluation information, we examined the relationship between the total number of times group members chose to rate their payoffs over the 100 rounds and the group's overall mean score (see Figure 4B). Figure 4B also displays each group's total number of exploratory choices over the 100 rounds. As can be seen, more evaluation was associated with lower group score (*r*(14)  = −0.613, *p* = 0.0198), indicating that evaluation information may indeed have had detrimental effects. Group score was also negatively correlated with the total number of exploratory choices (*r*(14)  = −0.854, *p* = 9.95 * 10^−5^). A causal analysis using a hierarchical Bayesian logit model with the MCMC method (see Methods section) revealed that evaluations and exploratory choices had a circular relation, whereby more evaluations led participants to engage in more exploratory behaviors, which facilitated further evaluations. This behavioral loop led participants in the frequency-plus-evaluation sub-condition to engage in over-exploration and fail to exploit better options for enough time (see Table S3 for details of the MCMC results).

### Fragility of evaluation information

**Figure 5:**
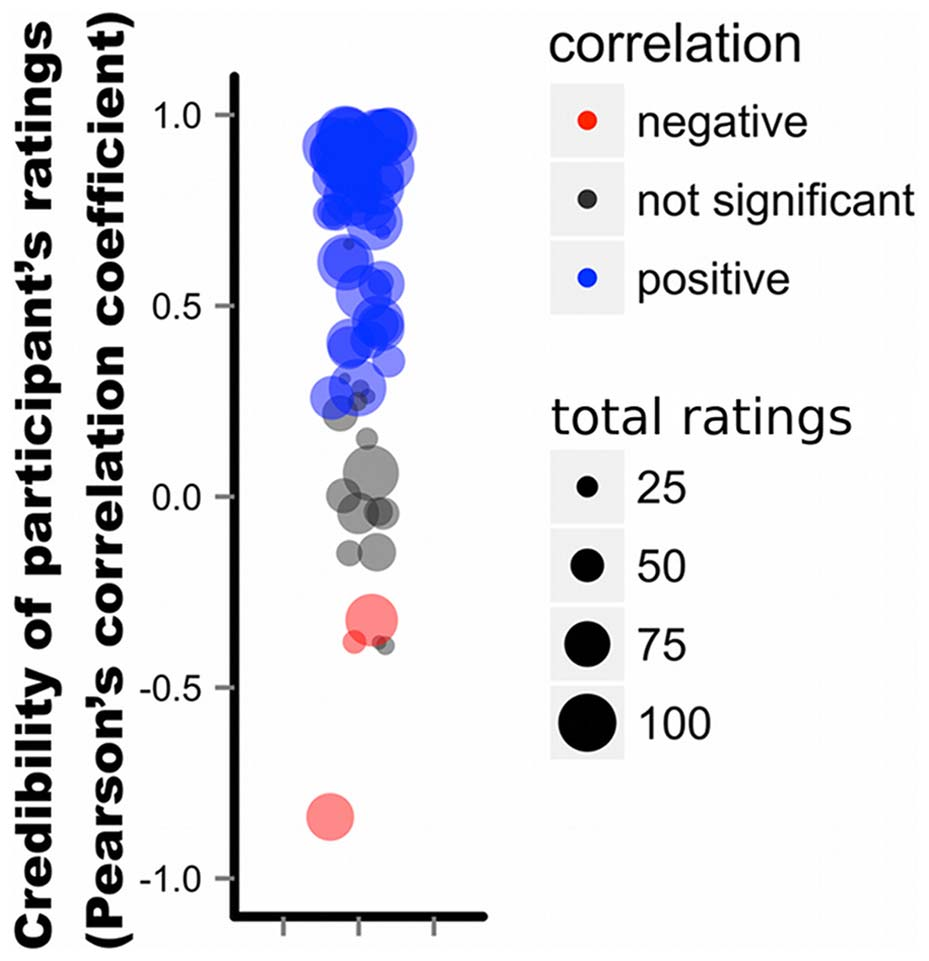
Credibility of evaluation information. The plot shows validities of each participant's evaluation information in the frequency-plus-evaluation sub-condition (N = 70). Each participant is represented by a circle. The y-axis refers to the Pearson's correlation coefficient between a participant's ratings of options on the 5-point scale and actual earned payoffs. The size of each circle represents the total number of ratings that the participant provided out of the 100 rounds. To reduce overlap, the circles were jittered horizontally.

As argued earlier, one possible reason for the detrimental effects of the evaluation information was that it could be faked much more easily than the frequency information. Figure 5 displays the validities of each participant's evaluation information in terms of correlation coefficients between the participant's ratings of options on the 5-point scale and actual experienced outcomes. As can be seen, 20 out of 70 participants in the frequency-plus-evaluation sub-condition provided invalid evaluations of options (i.e., correlations between their ratings of options and actual experienced payoffs were either non-significant or significantly negative). Three of them even rated bad options as “good” and/or good options as “bad”, suggesting strategic manipulations of evaluation.

**Figure 6:**
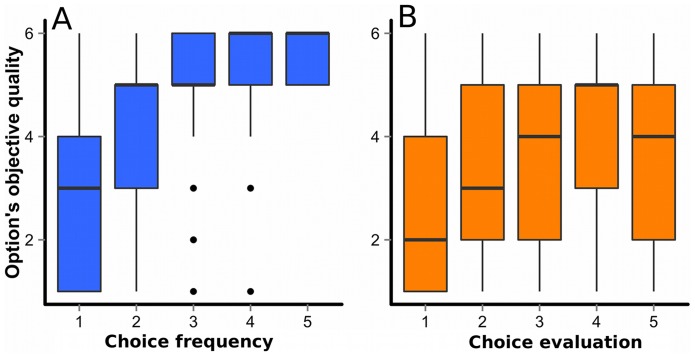
Validity of frequency and evaluation information in the frequency-plus-evaluation sub-condition. A: Relation between option quality and choice frequency. The y-axis refers to the mean objective quality of options chosen by 1, 2, 3, 4, or 5 participants in each round. B: Relation between option quality and choice evaluations. The y-axis refers to mean objective qualities of options rated on the 5-point scale ranging from 1 (very bad) to 5 (very good). We merged all rounds of all 14 groups into one dataset, thus both graphs represent 5000 observations each. The horizontal bars represent medians, while the boxes represent 50% ranges, and the vertical bars represent 1.5 * IQR (the inter-quartile range).

Finally, Figure 6A and 6B show overall validities of the two types of social information. Although both types were positively correlated with the objective option-qualities (frequency: *r* = 0.45, *p* = 2.2 * 10^−16^; evaluation: *r* = 0.22. *p* = 2.2 * 10^−16^), the correlation coefficient was significantly lower for the evaluation than the frequency information (*z* = 13.39, *p* = 3.52 * 10^−41^). The lower validity of the evaluation information was caused by individual differences in use of the rating scale (see Methods section for details). Modal points that participants used most frequently on the 5-point scale were different from each other (*F*(69, 13)  = 2.88, *p* = 0.0378), as reflected in the broad 50% ranges in Figure 6B.

## Discussion

The “multi-armed bandit” (MAB) problem is a pervasive adaptive task for humans as well as non-human animals. Eusocial insects often show an impressive array of performances on the MAB task through evolved coordinated mechanisms that function at the group level. Different from their colonies, however, the exploration-exploitation dilemma exists both within- and between-individuals for human groups composed of non-kin. This study thus focused on how a group of human agents may solve such dual dilemmas collectively with the aid of social information about each other's behavior.

Results from the experiment showed that average choice performance was substantially better in the group condition than in the individual condition (see Figure 2A). Analysis of choice behaviors suggest that on average, participants in the group condition were able to shorten total exploration time and exploit better options for a longer time by referring to each other's past behaviors (see Figure 2B). Interestingly, such exploratory costs were not borne evenly by all members – some members behaved as information scroungers [27], [28], [32] who free-rode on other members' exploratory efforts via social learning, harvested the “greedy” option [10] most of the time (see Figure 3) and earned more profits than information producers within each group (see Figure S4). However, opportunities to share social information were still beneficial for producing collective intelligence, which suggests that a group of human agents working on the MAB task can deal with the within- and between-individual dilemmas successfully, if not perfectly. In other words, social learning opportunities can provide greater net benefits to individuals above and beyond asocial learning, which is consistent with recent arguments from evolutionary theories emphasizing individual-fitness advantages of cultural learning [29], [32]–[34], [42]–[44].

For better performance in the MAB problem, it is important to switch from the exploration phase to the exploitation phase at some point and harvest from a reasonably good option, rather than keep exploring for the very best option all the time. In this sense, our study introduces an important dimension for the rapidly growing literature on human collective intelligence. It has been argued that social influence sometimes undermines collective performance on simple estimation tasks [45]. For example, in a recent study [24], each participant was first asked to make numeric estimates about some factual questions (e.g., population density of Switzerland, crime statistics), and then provided social information about other participants' estimates to reconsider her/his initial responses. Compared to the initial estimates, estimates after the social information were found to be less accurate, by which the researchers concluded that even mild social influence could undermine the wisdom of crowds effect. Although such demonstrations are useful in illuminating the functions of social influence, we conjecture that the experimental paradigm that focuses only on accuracy may miss a critical aspect of natural decision-making – cost-benefit trade-offs under a fixed time/energy budget. Given that most natural decisions are made under time/energy constraints, striking an optimal balance between the benefits of accuracy and necessary cognitive/physical costs is essential to maximizing the overall profitability of choices. By extending the MAB problem to a group context, this study has demonstrated that the benefit of social information arises not only from improving individual decision accuracies per se, but also from enabling agents to save exploration costs collectively, even in the face of the between-individual exploration-exploitation dilemma.

This study also examined how the richness of social information may affect collective performance. Contrary to the statistical intuition that more predictors should lead to better choices, sharing evaluations of options in addition to their frequency of choice had a detrimental effect on choice performance (see Figure 4A and 4B). Even though frequency information and evaluation information were both expected to be useful cues to predicting objective option-qualities, participants performed worse when both cues were combined than when only the frequency information was available, similar to the “less-is-more” effect in the judgment and decision making literature [46]. Our results suggest that at least two factors were responsible for this decline. First, nearly 30% of the participants seemed to try to manipulate others toward exploration of new options by providing unreliable evaluations (see Figure 5). Notice that for such manipulative purposes, faking evaluation is much cheaper and easier than faking the frequency information, which would require sacrificing the benefits of one's own behavioral choices. Second, participants used the rating scale differently from each other, especially in which of the 5 rating levels to use most frequently in evaluation. With such idiosyncratic individual biases, the average of those ratings across individuals could be misleading (see Figure 6A and 6B). In short, the evaluation information seems to be susceptible to manipulations and individual biases in scale-use, and thus more fragile than the social frequency information.

Given these results, it is tempting to argue that websites such as Amazon or Yelp that implement similar rating systems may also suffer from these problems. However, users of these websites may not have strong incentives to deceive others into exploring. Furthermore, rather than the 5-star rating system implemented in our study, many recent websites (the most famous being Facebook) have adopted a “Like” button where only positive evaluations are allowed. Interestingly, in the animal kingdom, most eusocial insects use only positive signals to recruit other workers to valuable resource patches (e.g., waggle dances by honeybees [15], pheromone trails [47] or tandem-running by ants [17]), while negative or mixed signals that can inform others of the “badness” of patches are rather rare. Given their impressive choice performance as colonies, it seems important to examine how these positive-negative differences in rating systems may affect group dynamics under uncertainty. Comparing different species from insect colonies to human societies and examining similarities and differences in their computational algorithms will be helpful in illuminating such questions [19], [22], [48], [49]. In future research, collaborations between biologists and social scientists on these topics will be essential for a better understanding of the nature of collective intelligence, which is much desired in our rapidly connecting societies.

## Materials and Methods

### Ethics statements

This study was approved by the Institutional Review Board of the Center for Experimental Research in Social Sciences at Hokkaido University (No. H24-3). Written informed consent was obtained from all participants before beginning the task.

### Participants

One hundred and ninety-four undergraduates (42 females; age: mean ± S.D.  = 19.8±1.44) were randomly selected from a subject pool at Hokkaido University in Japan to participate in the experiment. After the experiment, participants received monetary rewards based on their performance in the MAB task as compensation for their participation (mean ± S.D.  = 1175.7±139.6 JPY).

### Experimental procedure

For each experimental session, six to eight participants were brought into the laboratory. Five were randomly assigned to the group condition and the remaining participants were assigned to the individual condition. Upon arrival, each participant was seated in a separate semi-soundproof cubicle equipped with a computer terminal, and received further instructions individually on a computer screen. Participants remained strictly anonymous to each other throughout the experiment, and the monetary reward was also paid individually after the experiment. The experimental task software was constructed using z-Tree [50]. The entire session lasted about 75 minutes.

#### The MAB task

The experimental task assigned to participants was a 30-armed bandit problem in which they had to choose between 30 options that could yield different payoffs. Participants worked on the MAB task for a total of 100 rounds, and each round consisted of two stages: a choice stage followed by a feedback stage. First, each participant was asked to choose from an array of 30 options (see Figure 1) by clicking their preferred option. Next, participants were informed about their payoffs. Payoff amounts depended on the quality of the option each participant chose. For each chosen option, payoffs were randomly generated from a stationary uniform probability distribution with an interval [*min*, *min* +150] (where *min* is the option's minimum payoff), rounded up to the next integer. To simulate some foraging environments in which qualities of patches are negatively correlated with their frequency (i.e., high quality patches are rare), we varied the profitability of the 30 options as follows. Eleven of the 30 options were in the lowest-quality category (category 1) with *min*  = 0, eight options in category 2 with *min*  = 15, five options in category 3 with *min*  = 30, three options in category 4 with *min*  = 45, two options in category 5 with *min*  = 60, and only one option was in the highest-quality category (category 6) with *min*  = 75. The locations of these options on the computer screen were fixed across 100 rounds and were common for all participants in the same experimental session, but were randomly re-shuffled across different experimental sessions. At the outset of each session, participants were told that (a) they would have multiple (unspecified) choice opportunities in the experiment, (b) payoffs from their choices would be randomly determined by some unknown probability distribution unique to each option, and (c) those individual payoffs would be summed, multiplied by 0.1, and rounded up to the nearest ten Yen to determine their individual rewards after the experiment (see Figure S1 and S2 for more details about the program).

Thus, at the beginning, participants had no knowledge about the properties of the options, the exact shapes of the payoff distributions, and the total number of rounds. As time passed, however, participants could learn about the task structure via the feedback from their choice outcomes in each round.

#### Conditions

In addition to the private payoff-feedback, participants in the group condition could learn other members' choice behaviors in the preceding round. Two sub-conditions were nested in the group condition. Half of the 28 groups were randomly assigned to the “frequency only” sub-condition in which, at the choice stage, each participant could learn how many group members had chosen each option in the preceding round (see Figure 1A). The remaining 14 groups were assigned to the “frequency plus evaluation” sub-condition where, in addition to the abovementioned social-frequency information, participants could learn the average ratings that participants had given to their chosen options in the feedback stage (see Figure 1B). Rating the chosen option at the feedback stage was entirely optional, and participants could skip the evaluation and proceed to the next round. Contributing or skipping evaluation had no monetary impact. When all 5 members finished the feedback stage, they proceeded to the next choice stage. As a control, we also implemented an individual condition, in which 54 participants worked on the 30-armed bandit problem alone, without any social information.

### Statistical Analysis

#### Comparing participants' performance in the individual and group conditions

We used the qualities (category 1–6) of each option to score participants' choice performance in the MAB task. Because the difference in expected payoffs between any two adjacent categories were held constant (15 points), the qualities of options can be treated as an interval scale. We summed participant's choices over the 100 rounds, which yielded scores that could range from 100 (always choosing one of the 11 lowest-quality options) to 600 (always choosing the highest-quality option). Because participants in the group condition were nested in the same 5-person groups, we used a hierarchical linear model with the individual vs. group condition as a fixed effect and group-specific effect as a random effect to analyze their performance.

#### Comparing participants' exploratory choice frequencies between conditions

We used hierarchical Bayesian logit models with the Markov Chain Monte Carlo (MCMC) method to estimate per-individual exploration probability in the two conditions (equation S1) and in the two sub-conditions (equation S2) [51]. The individual vs. group condition, and the frequency-only vs. frequency-plus-evaluation sub-condition were treated as fixed effects, and individual- and group-specific effects were treated as random effects. We then examined the 95% Bayesian credible interval of a parameter (*λ*_1_) for the condition (or sub-condition) effect to see if the interval contained zero; if not, we interpreted the effect as significant. The MCMC simulation was conducted using the package rjags under R 3.0.3 and JAGS v 3.3.0 (further technical details are provided in the Supporting Information).

#### Comparing participants' performance in the frequency-only and frequency-plus-evaluation sub-conditions

We divided the 100 rounds into 5 blocks, and calculated each participant's average score in each block. Because the activities of participants in the same group were correlated, we averaged all 5 members' scores in each group to derive aggregate scores and assure independence of samples. Therefore, the unit of analysis was the group, with N = 28. We applied a 2 (Sub-conditions) × 5 (Blocks) repeated-measures ANOVA (available in the package “anovakun v.4.3.3” under R 3.0.1) to this data set.

#### Causal analysis of the relation between evaluation and exploration

We constructed two mixed logit models treating both individual- or group-specific effects as random effects. One model posits that an individual's exploration probability at round t+1 is influenced by the total number of ratings contributed in the group at round t (evaluation effect model; equation S3). The other, converse model assumes that probability of each member's contributing rating in the feedback stage at round t is influenced by whether or not she/he engaged in exploration in the choice stage at round t (exploration effect model; equation S4). We used the hierarchical Bayesian method [51] to infer the parameters using the package rjags under R 3.0.3 and JAGS v 3.3.0 (further technical details of this analysis are provided in *Supplementary* methods, Text S1). We used 95% credible intervals to determine the significance of each parameter.

#### Analysis of individual differences in scale-use

We first examined the modal point that each participant used in evaluation (i.e., which point of the 5-point scale, from 1 to 5, was used most frequently by each participant). We then applied a variance-ratio test to see whether the modes were more variable between participants belonging to the same group (as an index of individual idiosyncratic biases in scale-use) than between-group means.

### Supplementary data

The data is available in the Supporting Information, Data S1.

## Supporting Information

Figure S1 **Time sequence in the frequency only sub-condition.** At the choice stage of each round, each participant chose one of 30 green box icons, then continued to the feedback stage where she/he learned how many points they earned in this round. They continued to the next round's choice stage by clicking the “Next” button. Participants played 100 rounds in total, although they were not informed of this ahead of time.
(TIF)

Figure S2 **Time sequence in the frequency-plus-evaluation sub-condition.** At the feedback stage of each round, participants were asked to decide whether or not to contribute an evaluation of their chosen option. If yes, they were asked to rate the option on a 5-point scale before proceeding to the next round's choice stage.
(TIF)

Figure S3 **Trajectories of all participants**' **choices, shown separately for each group.** The performances of all 5 participants in each group are displayed in separate curves in each subplot. The y-axis refers to the objective quality (category) of the chosen options. The left column shows choice trajectories in the frequency-only sub-condition and the right column shows the frequency-plus-evaluation sub-condition. The numbers on the right indicate the rank of the group in terms of the average score of its 5 members, within the respective sub-condition. As can be seen, choices fluctuated more (i.e., were more exploratory) in the frequency-plus-evaluation sub-condition than in the frequency-only sub-condition, and more among lower ranked groups than among higher ranked groups.
(TIF)

Figure S4 **Relation between exploration frequency and total score.** The x-axis shows a participant's rank within their 5-person group in terms of exploration frequency. The y-axis refers to the participant's rank in terms of total score. The size of circle represents the total number of participants having the indicated rank combination.
(TIF)

Table S1 **MCMC results of the exploration probability model (equation S1).**
(PDF)

Table S2 **MCMC results of the exploration probability model (equation S2).**
(PDF)

Table S3 **MCMC results of the causality between exploration and evaluation.**
(PDF)

Data S1 (CSV)

Text S1 **Supporting methods.**
(DOCX)

## References

[1] Robins H (1952) Some aspects of the sequential design of experiments. Bull Am Math Soc 58: 527–535.

[2] Gittins J, Glazebook K, Weber R (2011) Multi-Armed Bandit Allocation Indices 2nd Edition. UK: John Wiley & Sons Ltd. 293 p.

[3] Kathehakis MN, Robbins H (1995) Sequential choice from several populations. Proc Natl Acad Sci USA 92: 8584–8585. 11607577 10.1073/pnas.92.19.8584 PMC41010

[4] Tewari A, Bartlett PL (2008) Optimistic linear programming gives Logarithmic regret for irreducible MDPs. In: Platt JC, Koller D, Singer Y, Roweis S, editors. Advances in Neural Information Processing Systems 20. Cambridge: MIT Press.

[5] Gittins JC (1979) Bandit processes and dynamic allocation indices. J Roy Stat Soc B 41: 148–177.

[6] Berry DA, Fristedt B (1985) Bandit Problems: Sequential Allocation of Experiments. Netherlands: Springer. DOI: 10.1007/978-94-015-3711-7.

[7] Brezzi M, Lai TL (2000) Incomplete learning endogenous data in dynamic allocation. *Econometrica* 68: 1511–1516.

[8] Keasar T, Rashkovich E, Cohen D, Shmida A (2002) Bees in two-armed bandit situations: foraging choices and possible decision mechanisms. Behav Ecol 13: 757–765.

[9] Anderson CM (2012) Ambiguity aversion in multi-armed bandit problems. Theor Decis 72: 15–33.

[10] Sutton RS, Barto AG (1998) Reinforcement Learning: An Introduction. Cambridge: MIT press.

[11] Tokic M, Palm G (2011) Value-difference based exploration: adaptive control between epsilon-greedy and softmax. In: KI 2011: Advances in Artificial Intelligence: 34th Annual German Conference on AI, Berlin, Germany, October 4–7, 2011, Proceedings. Heidelberg: Springer. 335–346.

[12] Auer P, Cesa-Bianchi N, Fischer P (2002) Finite-time analysis of the multi-armed bandit problem. Mach Learn 47: 235–256.

[13] Daw ND, O'Doherty JP, Dayan P, Seymour B, Dolan RJ (2006) Cortical substrates for exploratory decisions in humans. Nature 441: 876–879. 16778890 10.1038/nature04766 PMC2635947

[14] Cohen JD, McClure SM, Yu AJ (2007) Should I stay or should I go? How the human brain manages the trade-off between exploitation and exploration. Philos T Roy Soc B 362: 933–942. 10.1098/rstb.2007.2098 PMC2430007 17395573

[15] Seeley TD, Camazine S, Sneyd J (1991) Collective decision-making in honey bees: how colonies choose among nectar sources. Behav Ecol Sociobiol 28: 277–290.

[16] Czaczkes TJ, Grüter C, Jones SM, Francis LWR (2011) Synergy between social and private information increases foraging efficiency in ants. Biol Letters 7: 521–524. 10.1098/rsbl.2011.0067 PMC3130237 21325309

[17] Shaffer Z, Sasaki T, Pratt SC (2013) Linear recruitment leads to allocation and flexibility in collective foraging by ants. Anim Behav 86: 967–975.

[18] Detrain C, Deneubourg JL (2013) Collective decision-making and foraging patterns in ants and honeybees. Adv Insect Physiol 35: 123–173.

[19] Kameda T, Wisdom T, Toyokawa W, Inukai K (2012) Is consensus-seeking unique to humans? A selective review of animal group decision-making and its implications for (human) social psychology. Group Processes Interg 15: 673–689.

[20] Galton F (1907) Vox Populi. Nature 75: 450–451.

[21] Surowiecki J (2004) The Wisdom of Crowds: Why the Many Are Smarter than the Few and How Collective Wisdom Shapes Business, Economies, Societies and Nations. New York: Doubleday Books. 336 p.

[22] Conradt L, Roper TJ (2005) Consensus decision making in animals. Trends Ecol Evol 20: 449–456. 16701416 10.1016/j.tree.2005.05.008

[23] Wolf M, Kurvers RHJ, Ward AJW, Krause S, Krause J (2013) Accurate decision in an uncertain world: collective cognition increases true positives while decreasing false positives. Proc R Soc B 280: 20122777. 10.1098/rspb.2012.2777 PMC3574371 23407830

[24] Lorenz J, Rauhut H, Schweitzer F, Helbing D (2011) How social influence can undermine the wisdom of crowds effect. Proc Natl Acad Sci USA 108: 9020–9025. 21576485 10.1073/pnas.1008636108 PMC3107299

[25] Bolton P, Harris C (1999) Strategic experimentation. Econometrica 67: 349–374.

[26] Kameda T, Tsukasaki T, Hastie R, Berg N (2011) Democracy under uncertainty: the wisdom of crowds and the free-rider problem in group decision making. Psych Rev 118: 76–96. 10.1037/a0020699 20822292

[27] Giraldeau L-A, Caraco T (2000) Social Foraging Theory. Princeton: Princeton University Press. 376 p.

[28] Dubois F, Morand-Ferron J, Giraldeau L-A (2010) Learning in a game context: strategy choice by some keeps learning from evolving in others. Proc R Soc B 277: 3609–3616. 10.1098/rspb.2010.0857 PMC2982243 20573623

[29] Rogers AR (1988) Does Biology Constrain Culture? Am Anthropol 90: 819–831.

[30] Galef BGJ, Giraldeau L (2001) Social influences on foraging in vertebrates: causal mechanisms and adaptive functions. Anim Behav 61: 3–15. 11170692 10.1006/anbe.2000.1557

[31] Kameda T, Nakanishi D (2002) Cost-benefit analysis of social/cultural learning in a nonstationary uncertain environment: An evolutionary simulation and an experiment with human subjects. Evol Hum Behav 23: 373–393.

[32] Kameda T, Nakanishi D (2003) Does social/cultural learning increase human adaptability? Rogers's question revisited. Evol Hum Behav 24: 242–260.

[33] Enquist M, Eriksson K, Ghirland S (2007) Critical social learning: A solution to Rogers's paradox of nonadaptive culture. Am Anthropol 109: 727–734.

[34] Rendell L, Fogarty L, Laland KN (2009) Rogers' paradox recast and resolved: population structure and the evolution of social learning strategies. Evolution 64: 534–548. 19674093 10.1111/j.1558-5646.2009.00817.x

[35] Kameda T, Tamura R (2007) “To eat or not to be eaten?” Collective risk-monitoring in groups. J Exp Soc Psychol 43: 168–179.

[36] Krause J, Ruxton GD, Krause S (2009) Swarm intelligence in animals and humans. Trends Ecol Evol 25: 28–34. 19735961 10.1016/j.tree.2009.06.016

[37] Salganik MJ, Dodds PS, Watts DJ (2006) Experimental study of inequality and unpredictability in an artificial cultural market. Science 311: 854–856. 16469928 10.1126/science.1121066

[38] Muchnik L, Aral S, Taylor SJ (2013) Social influence bias: a randomized experiment. Science 341: 647–651. 23929980 10.1126/science.1240466

[39] McElreath R, Lubell M, Richerson PJ, Waring TM, Baum W, et al (2005) Applying evolutionary models to the laboratory study of social learning. Evol Hum Behav 26: 483–508.

[40] McElreath R, Bell AV, Efferson C, Lubell M, Richerson PJ, et al (2008) Beyond existence and aiming outside the laboratory: estimating frequency-dependent and pay-off-biased social learning strategies. Philos T Roy Soc B 363: 3515–3528. 10.1098/rstb.2008.0131 PMC2607339 18799416

[41] Toelch U, Bruce M, Meeus MTH, Reader SM (2010) Humans copy rapidly increasing choices in a multi-armed bandit problem. Evol Hum Behav 31: 326–333.

[42] Rendell L, Boyd R, Cownden D, Enquist M, Eriksson K, et al (2010) Why copy others? Insights from the social learning strategies tournament. Science 328: 208–213. 20378813 10.1126/science.1184719 PMC2989663

[43] Mesoudi A (2011) Cultural Evolution: How Darwinian Theory can Explain Human Culture and Synthesize the Social Sciences. Chicago, IL: University of Chicago Press. 280 p.

[44] Richerson P, Boyd R (2004) Not Genes Alone: How Culture Transformed Human Evolution. Chicago, IL: University of Chicago Press. 332 p.

[45] Asch S (1956) Studies of independence and conformity: a minority of one against a unanimous majority. Psychol Monogr-Gen A 70: 1–70.

[46] Gigerenzer G, Todd PM, ABC Research Group (1999) Simple Heuristics That Make Us Smart. Oxford: Oxford University Press. 416 p.

[47] Beckers R, Deneubourg JL, Goss S, Pasteels JM (1990) Collective decision making through food recruitment. Insect Soc 37: 258–167.

[48] Sumpter DJT (2010) Collective Animal Behavior. Princeton: Princeton University Press. 302 p.

[49] Krause J, Ruxton GD (2002) Living In Groups. Oxford: Oxford University Press. 210 p.

[50] Fischbacher U (2007) z-Tree: Zurich toolbox for ready-made economic experiments. Exp Econ 10: 171–178.

[51] Gelman A, Carlin JB, Stern HS, Dunson DB, Vehtari A, et al.. (2013) Bayesian Data Analysis, Third Edition. New York: CRC press. 661 p.

