# Supplementary figures and images for: Human Collective Intelligence under Dual Exploration-Exploitation Dilemmas
Source: PLoS One. 2014 Apr 22;9(4):e95789. doi: 10.1371/journal.pone.0095789 (PMC3995913; doi:10.1371/journal.pone.0095789)

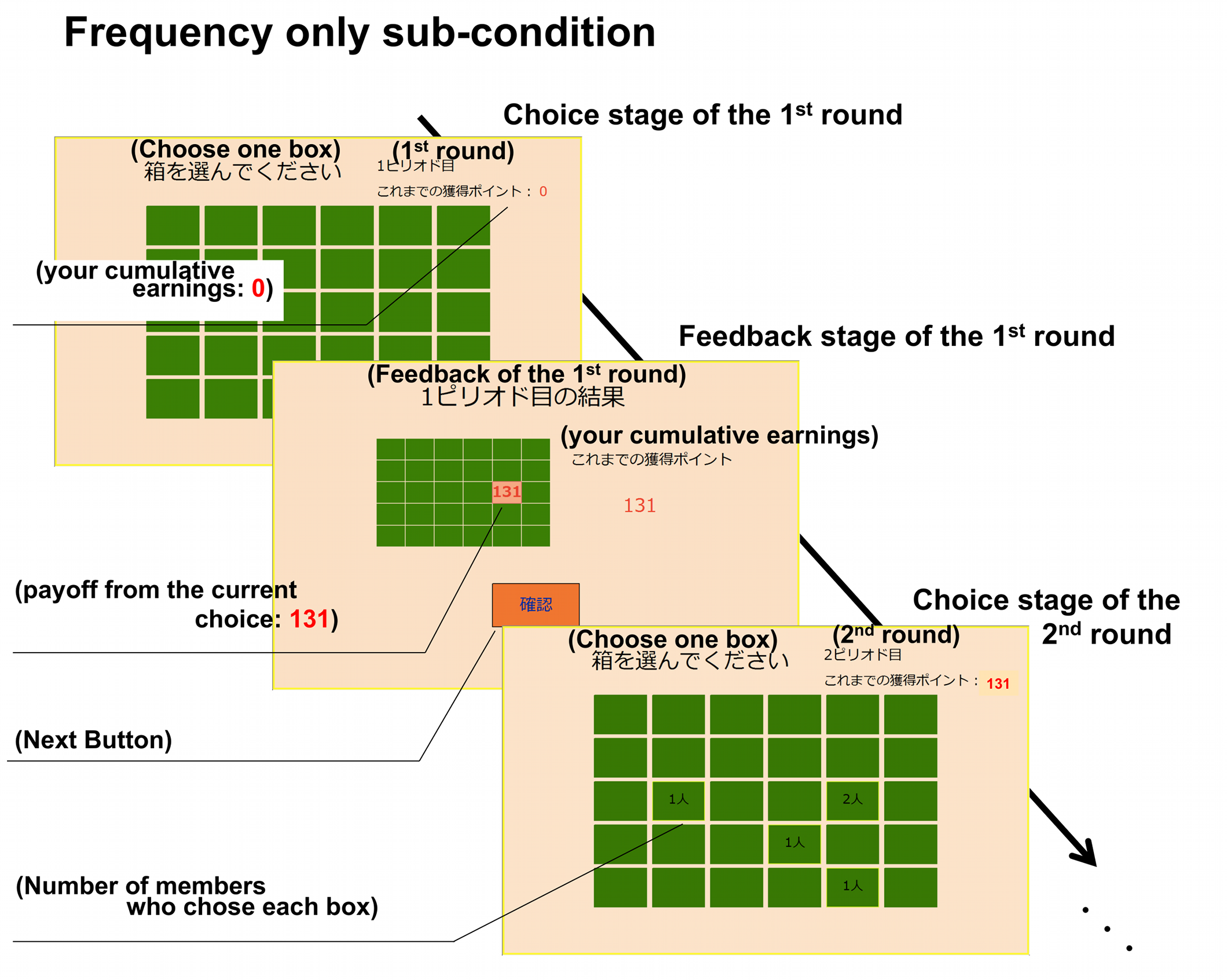

Supplement: Figure S1 — Time sequence in the frequency only sub-condition. At the choice stage of each round, each participant chose one of 30 green box icons, then continued to the feedback stage where she/he learned how many points they earned in this round. They continued to the next round's choice stage by clicking the “Next” button. Participants played 100 rounds in total, although they were not informed of this ahead of time. (TIF) [file pone.0095789.s001.tif]

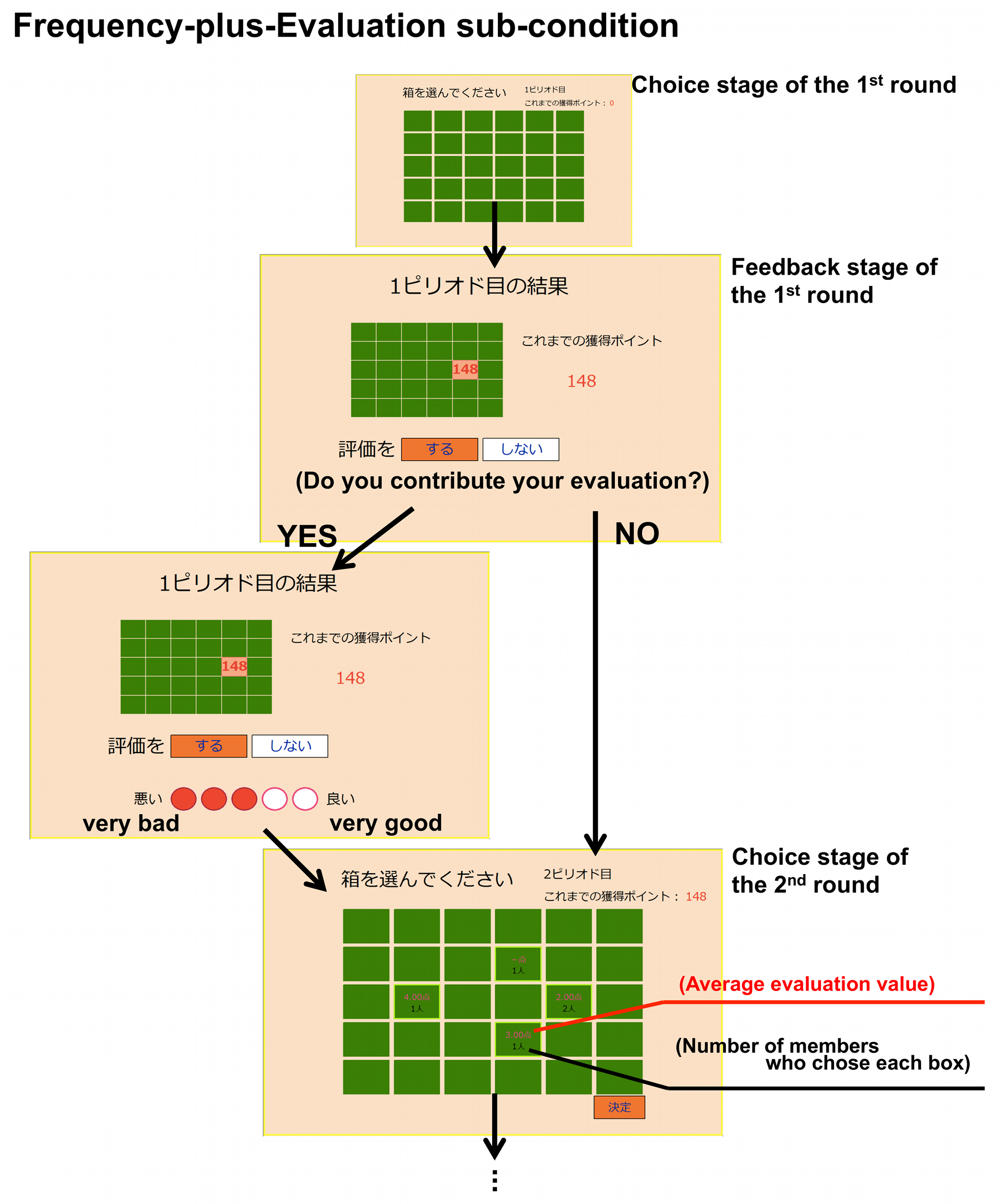

Supplement: Figure S2 — Time sequence in the frequency-plus-evaluation sub-condition. At the feedback stage of each round, participants were asked to decide whether or not to contribute an evaluation of their chosen option. If yes, they were asked to rate the option on a 5-point scale before proceeding to the next round's choice stage. (TIF) [file pone.0095789.s002.tif]

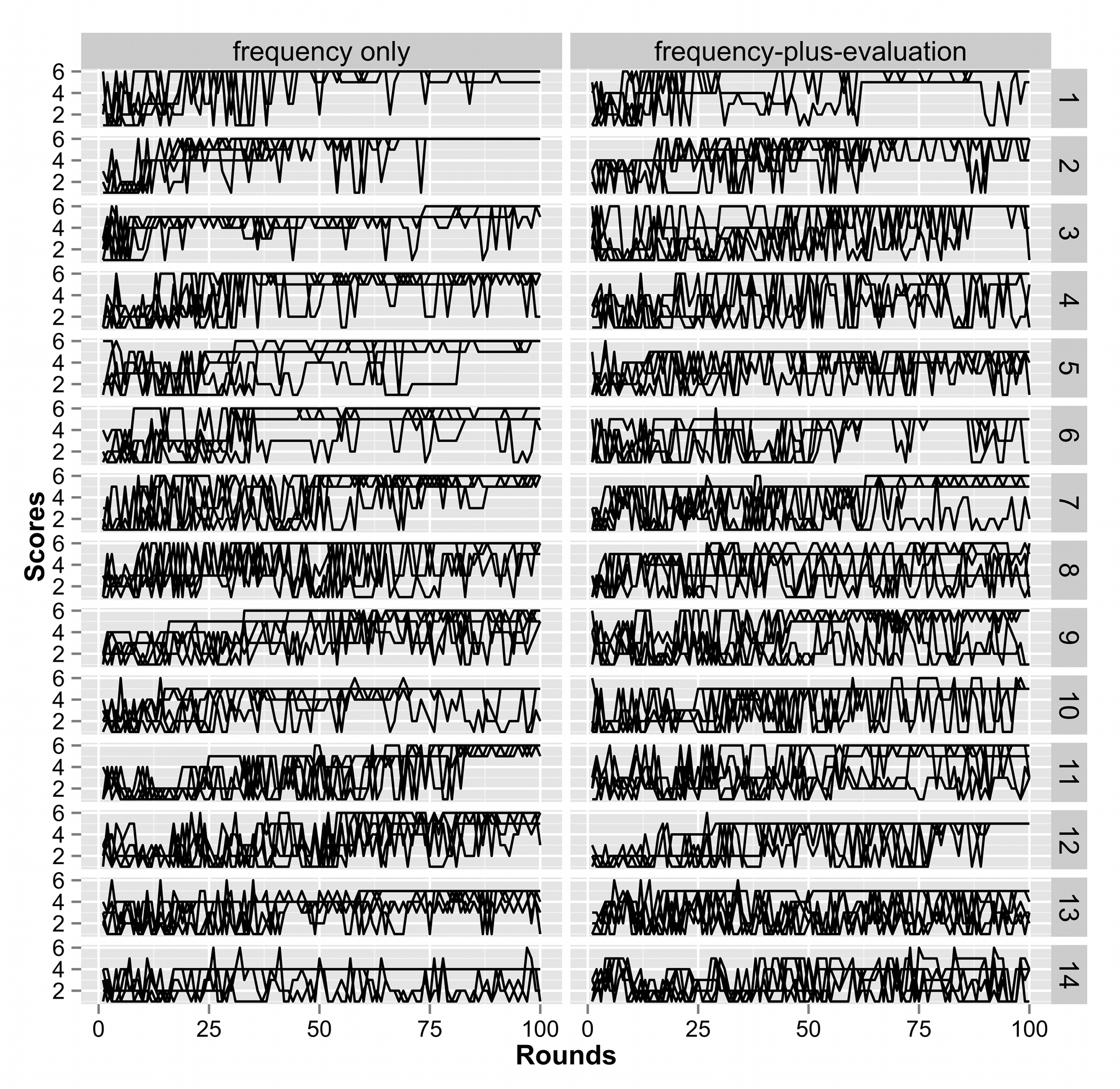

Supplement: Figure S3 — Trajectories of all participants' choices, shown separately for each group. The performances of all 5 participants in each group are displayed in separate curves in each subplot. The y-axis refers to the objective quality (category) of the chosen options. The left column shows choice trajectories in the frequency-only sub-condition and the right column shows the frequency-plus-evaluation sub-condition. The numbers on the right indicate the rank of the group in terms of the average score of its 5 members, within the respective sub-condition. As can be seen, choices fluctuated more (i.e., were more exploratory) in the frequency-plus-evaluation sub-condition than in the frequency-only sub-condition, and more among lower ranked groups than among higher ranked groups. (TIF) [file pone.0095789.s003.tif]

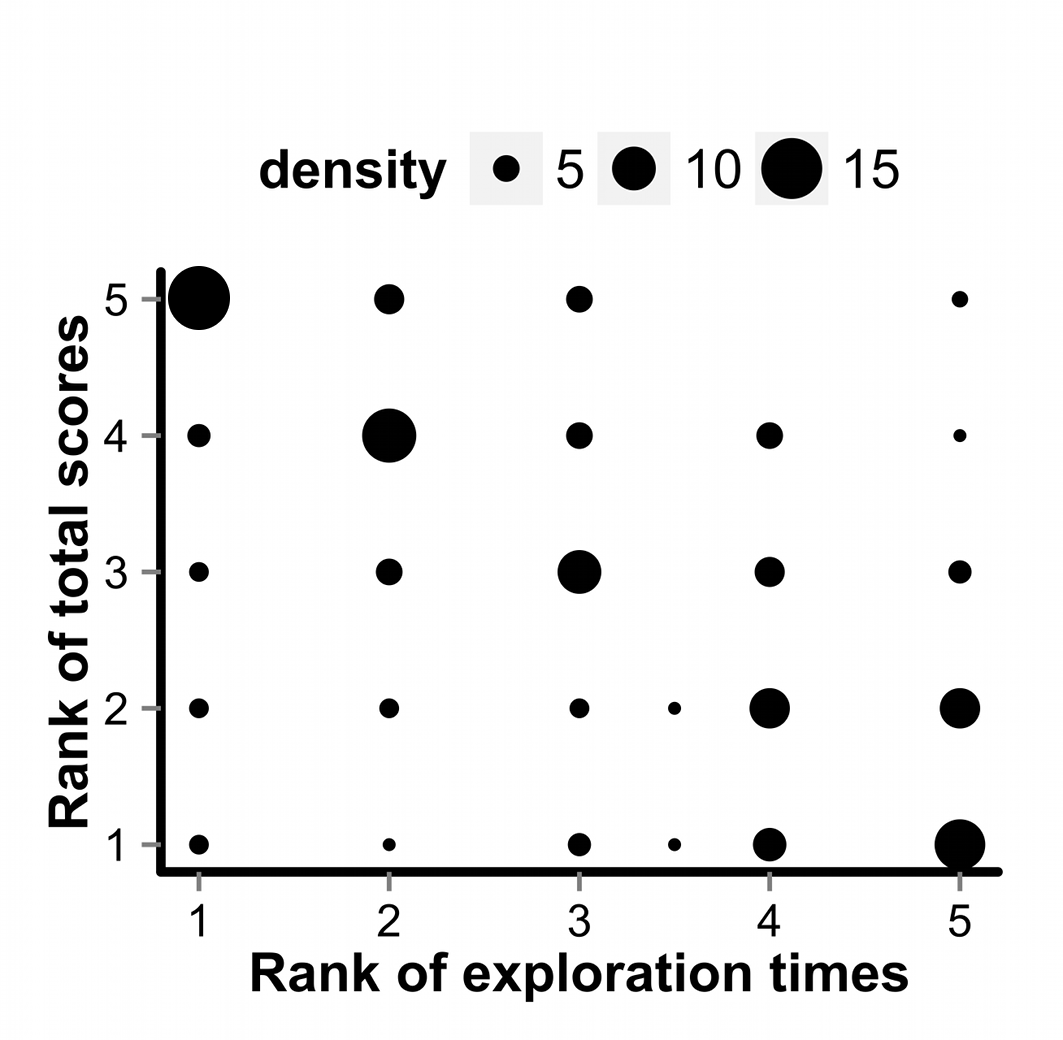

Supplement: Figure S4 — Relation between exploration frequency and total score. The x-axis shows a participant's rank within their 5-person group in terms of exploration frequency. The y-axis refers to the participant's rank in terms of total score. The size of circle represents the total number of participants having the indicated rank combination. (TIF) [file pone.0095789.s004.tif]
