# Supplementary material for: Human Collective Intelligence under Dual Exploration-Exploitation Dilemmas
Source: PLoS One. 2014 Apr 22;9(4):e95789. doi: 10.1371/journal.pone.0095789 (PMC3995913; doi:10.1371/journal.pone.0095789)
Supplement: Table S1 — MCMC results of the exploration probability model (equation S1). (PDF) [file pone.0095789.s005.pdf]

Table S1: MCMC results of the exploration probability model (equation S1)

Parameters	Mean	SD	Quantiles 2.5%	50.0%	97.5%	Gelman-Rubin Statistics median (upper C.I.)	Effective sample size
$\lambda_{1,0}$	1.94	0.25	1.42	1.94	2.47	1.00 (1.01)	1527
$\lambda_{1,1}$	-0.94	0.32	-1.63	-0.94	-0.27	1.00 (1.01)	1528
σ_1	1.62	0.11	1.42	1.61	1.86	1.00 (1.00)	4051
Σ_1	0.83	0.19	0.44	0.83	1.20	1.00 (1.01)	2921

The Gelman-Rubin statistic for each parameter was lower than 1.1, which means the MCMC sampling converged.
