# Supplementary material for: Human Collective Intelligence under Dual Exploration-Exploitation Dilemmas
Source: PLoS One. 2014 Apr 22;9(4):e95789. doi: 10.1371/journal.pone.0095789 (PMC3995913; doi:10.1371/journal.pone.0095789)
Supplement: Table S2 — MCMC results of the exploration probability model (equation S2). (PDF) [file pone.0095789.s006.pdf]

Table S2: MCMC results of the exploration probability model (equation S2)

Parameters	Mean	SD	Quantiles			Gelman-Rubin Statistics	
			2.5%	50.0%	97.5%	median (upper C.I.)	Effective sample size
$\lambda_{2,0}$	0.54	0.30	-0.05	0.54	1.13	1.01 (1.02)	1675
$\lambda_{2,1}$	0.92	0.43	0.07	0.91	1.78	1.00 (1.01)	1959
σ_2	1.70	0.13	1.42	1.69	1.96	1.00 (1.00)	3611
Σ_2	0.80	0.25	0.28	0.80	1.31	1.00 (1.00)	2494

The Gelman-Rubin statistic for each parameter was lower than 1.1, which means the MCMC sampling converged.
