# Supplementary material for: Human Collective Intelligence under Dual Exploration-Exploitation Dilemmas
Source: PLoS One. 2014 Apr 22;9(4):e95789. doi: 10.1371/journal.pone.0095789 (PMC3995913; doi:10.1371/journal.pone.0095789)
Supplement: Table S3 — MCMC results of the causality between exploration and evaluation. (PDF) [file pone.0095789.s007.pdf]

Table S3: MCMC results of the causality between exploration and evaluation

	Parameters	Mean	SD	Quantiles			Gelman-Rubin Statistics	
				2.5%	50.0%	97.5%	median (upper C.I.)	Effective sample size
Evaluation effect model (*equation S3*)	α_1	1.47	0.32	0.86	1.48	2.10	1.01 (1.03)	1000
	β_1	0.42	0.040	0.34	0.42	0.50	1.00 (1.02)	1241
	σ_3	1.93	0.20	1.57	1.91	2.38	1.01 (1.03)	1118
	Σ_3	0.67	0.39	0.04	0.66	0.90	1.00 (1.01)	814
Exploration effect model (*equation S4*)	α_2	0.87	0.39	0.094	0.86	1.67	1.01 (1.02)	614
	β_2	0.34	0.089	0.17	0.34	0.52	1.00 (1.01)	1167
	σ_4	2.09	0.25	1.67	2.06	2.60	1.00 (1.01)	995
	Σ_4	0.98	0.47	0.12	0.96	2.01	1.00 (1.02)	506

The Gelman-Rubin statistic for each parameter was lower than 1.1, which means the MCMC sampling converged.
