# Supplementary material for: Human Collective Intelligence under Dual Exploration-Exploitation Dilemmas
Source: PLoS One. 2014 Apr 22;9(4):e95789. doi: 10.1371/journal.pone.0095789 (PMC3995913; doi:10.1371/journal.pone.0095789)
Supplement: Text S1 — Supporting methods. (DOCX) [file pone.0095789.s009.docx]

Supporting Information

Human collective intelligence under dual exploration-exploitation dilemmas

Toyokawa, W., Kim, H. & Kameda, T.

Supporting methods

*Defining exploration and exploitation*

We followed the traditional definitions of exploration and exploitation in the reinforcement learning literature [1]. Individual *x* facing a choice at round *t* has a unique vector $Q\left( x, t \right)=\left\{ Q_{1, t}^{x}, Q_{2, t}^{x}, Q_{3, t}^{x}, \ldots, Q_{30, t}^{x} \right\}$, whose elements represent expected payoffs of the 30 options based on *x*’s cumulative knowledge up to round *t*. As *x*’s experience accumulates, *Q* (*x, t*) is updated by an averaging rule, in which is equal to the mean payoff that *x* has earned from option *i* until round *t*. The averaging updating rule is a suitable assumption of the action-value function in a stationary multi-armed bandit task [1]. This model assumes that players can hold up to 30 values for the different options simultaneously in working memory and calculate a new average for each option. Although such assumptions are too strong for human cognitive capacity, we believe that this model can provide a first approximation to distinguish between exploration and exploitation in a stationary multi-armed bandit task. We categorized choice behaviour as “exploitation” when the individual *x* chose option *i* with max in round *t* + 1 (i.e., the greedy choice). Otherwise, we categorized the choice as “exploration.” The initial *Q* values were set to zero, i.e., $Q\left( x, 0 \right)= \left\{ 0, 0, 0, \ldots, 0 \right\}$, because *x* had no prior knowledge about the expected payoff from each option. For the first round (*t* = 1) only, we categorized all choices as “exploration.”

*Comparing exploration frequencies between the individual and group conditions*

To compare the frequencies of exploratory choices over all 100 rounds between the individual and group conditions, we used a hierarchical Bayesian method. We modelled the probability of individual *x* in group $g$ engaging in exploration ($q_{x, g}$) as a mixed logit model:

$\ln\left( \frac{q_{x,g}}{1-q_{x,g}} \right)= \lambda_{1,0}+ \lambda_{1,1}*\left( condition \right)+ \varepsilon_{1}\left( x \right)+ E_{1}\left( g \right),$ (S1)

where λ_1, 0_ indicates an intercept, λ_1, 1_ indicates a fixed effect for the condition (dummy coded: individual condition = 0; group condition = 1), and local parameters *ε*_1_ (*x*) and $E_{1}\left( g \right)$ indicate random effects specific to the individual and to the group respectively ($\varepsilon_{1}\left( x \right) \sim Normal\left( 0, \sigma_{1} \right)$ and $E_{1}\left( g \right) \sim Normal\left( 0, \Sigma_{1} \right)$).

To estimate the four parameters (λ_1, 0_, λ_1, 1_, σ_1_, and Σ_1_), we set the uninformed priors of λ_1, 0_ and λ_1, 1_ to a normal distribution with mean = 0 and variance = 10^4^ (i.e., a very stretched distribution), and those of the hyper parameters (σ_1_ and Σ_1_) to a uniform distribution [0, 10^4^]. We conducted Markov Chain Monte Carlo (MCMC) simulations for 5 independent sequences. The number of total iterations per chain was 240,000 (thinning rate was 250), and first 40,000 steps were discarded from analysis because of initial-value dependencies. We used 95% Bayesian credible intervals to determine the significance of each parameter.

*Comparing exploration frequencies between the frequency-only and frequency-plus-evaluation sub-conditions*

To compare the frequencies of exploratory choices over all 100 rounds between the two sub-conditions, we used a hierarchical Bayesian method. We modelled the probability of individual *x* in group $g$ engaging in exploration ($q_{x, g}$) as a mixed logit model:

$\ln\left( \frac{q_{x,g}}{1-q_{x,g}} \right)= \lambda_{2,0}+ \lambda_{2,1}*\left( subcondition \right)+ \varepsilon_{2}\left( x \right)+ E_{2}\left( g \right),$ (S2)

where λ_2, 0_ indicates an intercept, λ_2, 1_ indicates a fixed effect for the sub-condition (dummy coded: *frequency-only* sub-condition = 0; *frequency-plus-evaluation* sub-condition = 1), and local parameters *ε*_2_ (*x*) and $E_{2}\left( g \right)$ indicate random effects specific to the individual and to the group respectively ($\varepsilon_{2}\left( x \right) \sim Normal\left( 0, \sigma_{2} \right)$ and $E_{2}\left( g \right) \sim Normal\left( 0, \Sigma_{2} \right)$).

Because the structure of the model was identical to the equation S1, the MCMC protocol was the same as described above.

*Analysis of the causality between exploration and evaluation*

To analyze factors affecting individual exploratory choices, we constructed two models. One model posits that an individual’s exploration probability at round *t* + 1 is influenced by the total number of ratings contributed in the group at round *t* (evaluation effect model). The converse model posits that the individual probability of contributing rating information at the feedback stage of round *t* is influenced by whether or not she/he has engaged in exploration at the choice stage of round *t* (exploration effect model).

For the evaluation effect model, the probability of individual *x* in group $g$ engaging in exploration at round *t* + 1 ($p_{x, g, t+1}$) was modelled:

$ln\left( \frac{p_{x, g, t+1}}{1-p_{x, g, t+1}} \right)=\alpha_{1}+\beta_{1}*W_{g, t}+\varepsilon_{3}\left( x \right)+E_{3}\left( g \right),$ (S3)

where $z_{\mathrm{ij}}=\beta1+\beta2*condition\left( i \right)+r\left( i \right)+rg\left( j \right),$α_1_ indicates an intercept, β_1_ indicates a fixed effect for the total number of ratings contributed in the group at the preceding round ($W_{g, t}$), and local parameters *ε*_3_(*x*) and $E_{3}\left( g \right)$ indicate random effects specific to the individual and to the group respectively ($\varepsilon_{3}\left( x \right) \sim Normal\left( 0, \sigma_{3} \right)$ and $E_{3}\left( g \right) \sim Normal\left( 0, \Sigma_{3} \right)$).

To estimate the four parameters (*α*_1_, *β*_1_, σ_3_, and Σ_3_), we set the uninformed priors of *α*_1_ and *β*_1_ to a normal distribution with mean = 0 and variance = 10^4^ (i.e., a very stretched uniform distribution), and those of the hyper parameters (*σ*_3_ and Σ_3_) to a uniform distribution [0, 10^4^]. We conducted MCMC simulations for 5 independent sequences. The number of total iterations per chain was 63,000 (thinning rate was 300), and first 3,000 steps were discarded from analysis because of initial-value dependencies. We used 95% credible intervals to determine the significance of each parameter.

For the exploration effect model, the probability of contributing evaluation at the feedback stage of round *t* was modelled:

$\ln\left( \frac{\omega_{x, g, t}}{1-\omega_{x, g, t}} \right)=\alpha_{2}+\beta_{2}*\left( exploration \right)_{x, t}+\varepsilon_{4}\left( x \right)+E_{4}\left( g \right),$ (S4)

where *ω_x,g,t_* indicates the probability of individual *x* contributing her/his evaluation at round *t*, α_2_ for an intercept, β_2_ for a fixed effect of exploration in the current period *t* (dummy coded: exploration = 1, exploitation = 0). Local parameters *ε*_4_(*x*) and *Ε*_4_(*g*) indicate random effects specific to the individual and to the group respectively ($\varepsilon_{4}\left( x \right) \sim Normal\left( 0, \sigma_{4} \right)$and $E_{4}\left( g \right) \sim Normal\left( 0, \Sigma_{4} \right)$). Because the structure of the model was identical to the evaluation effect model, the MCMC protocol was the same as described above.

References

[1] Sutton, R. S. & Barto, A. G. *Reinforcement Learning: An Introduction*. (MIT press, Cambridge, 1998).
